# Unfractionated heparin in acute chest syndrome: a pilot feasibility randomized controlled trial of unfractionated heparin vs. standard of care in acute chest syndrome

**DOI:** 10.1186/s40814-020-00715-w

**Published:** 2020-11-10

**Authors:** Craig D. Seaman, Enrico Novelli, Laura De Castro, Margaret V. Ragni

**Affiliations:** 1 Department of Medicine, Division of Hematology/Oncology, University of Pittsburgh Medical Center, Pittsburgh, PA USA; 2 Hemophilia Center of Western Pennsylvania, 3636 Boulevard of the Allies, Pittsburgh, PA USA

**Keywords:** Acute chest syndrome, Heparin, Sickle cell disease, Thrombosis

## Abstract

**Background:**

Acute chest syndrome (ACS) is the leading cause of mortality in sickle cell disease (SCD). The pathogenesis of ACS is complex and not entirely understood with multiple etiologies likely contributing simultaneously. One particular etiology is pulmonary vascular occlusion due to thrombosis. Thus, anticoagulation is an attractive therapeutic modality.

**Methods:**

This was a single-center, randomized controlled, open-label, pilot study to determine the feasibility of performing a larger multicenter phase III trial to assess the effects of unfractionated heparin (UFH) in ACS. Subjects were randomized within 24 h of diagnosis of ACS to one of two treatment arms, UFH, and standard of care (SOC), or no UFH and SOC. UFH was given intravenously for 7 days, or until discharge, if discharge was shorter than 7 days. SOC consisted of intravenous fluids, antibiotics, supplemental oxygen, analgesia, red blood cell transfusion, and exchange transfusion.

**Results:**

From July 2014 to June 2018, a total of 7 patients underwent randomization (four patients received UFH in addition to SOC and 3 patients received SOC only). Two of the prespecified feasibility criteria were not met: the capacity to consent eligible individuals and the timely notification of hospitalized patients with ACS necessary to permit randomization within 24 h of diagnosis; thus, as a result of poor enrollment, the study was terminated early. The duration of hospitalization was 279.43 (SD 267.98) and 127.31 (SD 137.70) h in the UFH and SOC arms, respectively. The duration of hypoxemia, leukocytosis, fever, and moderate to severe pain was 117.52 (SD 60.52), 24.90 (SD 29.69), 117.52 (SD 60.52), and 117.52 (SD 60.52) h, respectively, in the UFH group, and 51.49 (SD 44.79), 0, 53.11 (SD 25.06), and 88.68 (SD 72.77) h, respectively, in the SOC group. No major bleeding was noted in either group.

**Conclusions:**

Our study did not achieve prespecified feasibility criteria, resulting in poor enrollment and early termination, and serves to highlight some of the pitfalls experienced in clinical research in SCD. It did show the use of UFH without any major adverse events in 7 subjects. No future large-scale study is planned.

**Trials registration:**

Registered at ClinicalTrials.gov (NCT #02098993) on March 28, 2014.

## Key lessons on feasibility

- It would have been beneficial to involve patient representatives and have patients complete a pre-study feasibility survey whether or not they would be willing to participate in the study, in the event they were deemed a candidate.
- It would have been useful to have healthcare providers introduce the study to patients during regular outpatient clinic visits.
- It would have been helpful to identify a dedicated research coordinator intimately involved with the care of the patients.
- It would have been valuable to utilize the electronic medical record to ensure notification of potential subjects.

## Background

Sickle cell disease (SCD) is an autosomal recessive hemoglobinopathy caused by a glutamic acid to valine substitution at the sixth codon of the beta globin gene resulting in the production of mutant hemoglobin S that is prone to intraerythrocytic polymerization and subsequent microvascular occlusion clinically manifested as episodes of severe pain, organ damage, and early death [1, 2]. SCD affects approximately 75,000 to 100,000 African Americans in the USA [3]. The second leading cause of admission to the hospital and leading cause of mortality in SCD is acute chest syndrome (ACS), defined as a new pulmonary infiltrate associated with fever, chest pain, and signs and symptoms of pulmonary disease, such as cough, dyspnea, tachypnea, and hypoxemia [4]. Its clinical course can be unpredictable and optimal treatment remains largely based on expert opinion. Nearly half of all patients with SCD will develop ACS at least once with a smaller subset suffering multiple episodes [4]. Repeated episodes of ACS are associated with the development of chronic lung disease and early death [4].

The pathogenesis of ACS is complex and not entirely understood with multiple etiologies likely contributing simultaneously in any one particular episode. The most common etiologies of ACS include infection, infarction due to pulmonary vascular occlusion, and fat embolism [5]. Pulmonary vascular occlusion may be microvascular, due to thrombosis or sickled red blood cells, or occur in larger vessels as seen with pulmonary embolism (PE) [4]. Pulmonary vascular occlusion due to thrombosis is becoming increasingly more recognized as an important player in the pathogenesis of ACS. There is a 17% prevalence of pulmonary artery thrombosis in patients admitted to the hospital with ACS [6]. At autopsy of 21 SCD patients with sudden, unexpected death, 38.1% of patients had PE and 28.5% of patients had pulmonary microvascular thrombi, defined as < 1 ml in size and undetectable with current CT techniques [7]. Nearly every component of hemostasis, including platelet function and the procoagulant, anticoagulant, and fibrinolytic systems, is altered in the direction of the procoagulant phenotype, in SCD [8]. Specific mechanisms of hypercoagulability include the loss of normal membrane phospholipid asymmetry from repeated cycles of sickling and unsickling resulting in abnormal phosphatidylserine exposure, which is associated with increased levels of markers of thrombin generation and increased endothelial tissue factor expression [8]. Also, free plasma hemoglobin from hemolysis in SCD scavenges nitric oxide, which normally inhibits platelet activation and aggregation, resulting in chronic platelet activation [9].

Given the increasingly recognized role of thrombosis in SCD, including vasooclusive crises (VOC) and ACS, anticoagulation represents an attractive therapeutic modality. Unfractionated heparin (UFH), in addition to its anticoagulant effects, functions as a P-selectin antagonist and decreases sickle cell adhesion to the endothelium under static conditions as well as P-selectin mediated flow adherence of sickle cells to thrombin-treated human vascular endothelial cells [4]. A few studies have evaluated the use of low molecular weight heparin in SCD and VOC with varying results [10, 11]. However, to our knowledge, the use of anticoagulation in the treatment of ACS has not been assessed. So, we proposed a single-center, randomized controlled, open-label, pilot study to determine the feasibility of performing a larger multicenter phase III trial to assess the effects of UFH in ACS.

## Methods

### Study design

This was a single-center, randomized controlled, open-label, pilot study to determine the feasibility of performing a larger multicenter phase III trial to assess the effects of UFH in ACS. Initially, following the diagnosis of ACS, the principal investigator (PI) was notified about potential subjects by one of sickle cell disease healthcare providers. Subsequently, due to missed notifications, a dedicated research coordinator was engaged to notify the PI of potential subjects utilizing the electronic medical record (EMR) to ensure timely notification following the diagnosis of ACS. Subjects meeting inclusion and exclusion criteria, and consented by the PI to study participation, were randomized within 24 h of diagnosis of ACS to one of two treatment arms, anticoagulation, and standard of care (SOC), or no anticoagulation and SOC. Simple randomization with 1:1 allocation was performed by an independent statistician using a computerized random number generation and concealed in sequentially labeled envelopes. Weight-adjusted UFH was given at doses of 80 units per kilogram followed by 18 units per kilogram per hour intravenously for 7 days, or until discharge, if the discharge was shorter than 7 days. UFH was monitored by standard protocol to maintain the anti-Xa UFH level in the therapeutic range per institutional guidelines (Table 1). The standard of care was at the discretion of the treating physician, but guidance was given and consisted of intravenous fluids, antibiotics, supplemental oxygen, analgesia, red blood cell transfusion, and exchange transfusion (Table 2).

**Table 1:** Unfractionated heparin nomogram

Anti-Xa level	Dosing change	Repeat anti-Xa level
< 0.2 units per mL	80 unit per kilogram bolus (maximum 10,000 units) followed by an increase in the rate of the infusion by 4 units per kilogram per hour	Repeat anti-Xa level 6 h later
0.21–0.29 units per mL	40 unit per kilogram bolus (maximum 10,000 units) followed by an increase in the rate of the infusion by 2 units per kilogram per hour	Repeat anti-Xa level 6 h later
0.30–0.70 units per mL (therapeutic)	No changes necessary	Repeat anti-Xa level every 6 h until two consecutive therapeutic anti-Xa levels are achieved then check anti-Xa level daily
0.71–0.80 units per mL	Decrease the rate of the infusion by 2 units per kilogram per hour	Repeat anti-Xa level 6 h later
0.81–1.00 units per mL	Stop the infusion for 1 h then restart after decreasing the rate of infusion by 3 units per kilogram per hour	Repeat anti-Xa level 2 h later
> 1.00 units per mL	Stop the infusion for 2 h then restart after decreasing the rate of infusion by 4 units per kilogram per hour	Repeat anti-Xa level 2 h later

**Table 2:** Standard of care guidelines

Intervention	Comment
Intravenous fluids	Maintain adequate hydration with hypotonic crystalloids.
Antibiotics	Treat pathogens responsible for community acquired pneumonia, including “atypicals” or, if necessary, provide a broader spectrum of coverage against antimicrobial organisms.
Supplemental oxygen	Maintain arterial oxygen saturation greater than or equal to 95%.
Analgesia	Maintain adequate analgesia with specifics regarding pain management individualized according to each patient’s established plan of care as dictated by his or her outpatient hematologist.
Red blood cell transfusion	Administered as standard clinical practice in acute chest syndrome with the goal of preventing progression to acute respiratory failure.
Exchange transfusion	Recommended if extensive bilateral pulmonary disease is present, hypoxemia is not corrected with supplemental oxygen, or rapid clinical deterioration occurs.

### Study patients

Study patients consisted of adults with SCD (genotype HbSS, SC, or Sβ^0^), aged 18 years or older, admitted to one of four tertiary care University of Pittsburgh Medical Center hospitals with a diagnosis of ACS, which was defined as a new pulmonary infiltrate involving at least one segment of the lung on a chest X-ray or chest CT scan with 2 or more of the following: chest pain, tachypnea (defined as a respiratory rate greater than 20 breaths per minute), dyspnea, cough, hypoxemia (defined as oxygen saturation less than 90%), or body temperature greater than or equal to 38.0 °C. Exclusion criteria included any absolute contraindication to heparin, platelet count less than 50,000 per microliter, history of moyamoya disease or proliferative retinopathy, active participation in a chronic exchange transfusion program, underlying hypercoagulable disorder other than SCD, currently receiving therapeutic anticoagulation or antiplatelet agents, currently receiving estrogen-containing hormonal therapy, or chest CT scan-documented PE performed as standard of care prior to study enrollment.

### Feasibility criteria

Prespecified feasibility criteria included the following: the capacity to consent eligible individuals; the timely notification of hospitalized patients with ACS, which is necessary to randomize patients within 24 h of diagnosis; the proper administration of the study drug; and the ability to completely and accurately collect clinical data of interest.

### Clinical outcomes

In addition to exploring the feasibility criteria above, it was sought to provide preliminary data, with respect to treatment effect and variance, for future sample size calculations given the lack of existing data to help guide this process. The primary clinical outcome was the duration of hospitalization. Key secondary clinical outcomes included the duration of hypoxemia, fever, leukocytosis, and moderate to severe pain utilizing the visual analog scale for pain. Other secondary clinical outcomes included a total dose of opioids, number of red blood cell transfusions administered, transfer to the intensive care unit, the requirement for mechanical ventilation, and development of multiorgan failure syndrome. All clinical outcomes were defined as beginning with randomization and ending with resolution or study completion (Table 3). Safety outcomes consisted of allergic reaction, bleeding, thrombocytopenia, and heparin-induced thrombocytopenia.

**Table 3:** Outcome definitions

Outcome	Outcome definition	Onset	Resolution
Duration of hospitalization (measured in hours)	Duration of hospitalization	Randomization	Discharge from hospital
Duration of hypoxemia (measured in hours)	Arterial oxygen saturation less than 90%	Randomization	Failure to meet criteria for hypoxemia on 3 consecutive assessments or study completion
Duration of fever (measured in hours)	Body temperature greater than or equal to 38.0 °C	Randomization	Failure to meet criteria for fever on 3 consecutive assessments or study completion
Duration of leukocytosis (measured in hours)	White blood cell count greater than 10,000 per liter	Randomization	Failure to meet the criteria for leukocytosis on 2 consecutive assessments or study completion
Duration of moderate to severe pain (measured in hours)	Score of 4 or greater on the VAS for pain	Randomization	Score of less than 4 on the VAS for pain on 2 consecutive assessments or study completion
Total dose of opioids	Measured in milligram of oral morphine equivalents	Randomization	Study completion
Number of red blood cell transfusions	Number of red blood cell transfusions	Randomization	Study completion
Transfer to the intensive care unit	Yes or no	Randomization	Study completion
Mechanical ventilation	Yes or no	Randomization	Study completion
Multiorgan failure syndrome	Acute development of 2 or more organs or organ systems unable to maintain homeostasis in a critically ill individual	Randomization	Study completion

*VAS* visual analog scale

### Statistical analysis

A goal sample size of 20 subjects for the pilot study was based on clinical rather than statistical considerations to provide a preliminary estimate of primary and secondary clinical outcomes. Based on 75,000 to 100,000 African Americans with SCD in the USA who develop ACS at a frequency of 8.8 per 100 patient years, with 50% developing ACS at some point during his or her lifetime, and given the fact that approximately 30 patients are admitted annually at participating UPMC hospitals with ACS, it was estimated that at least 80%, or 24 patients per year would meet inclusion and exclusion criteria, and anticipated enrolling 10 subjects, or slightly less than 50% per year. This was a pilot study, so a descriptive statistical analysis was planned, including frequencies and percentages for categorical variables and mean, range, and standard deviation for continuous variables.

## Results

### Patient characteristics

**Fig. 1:**
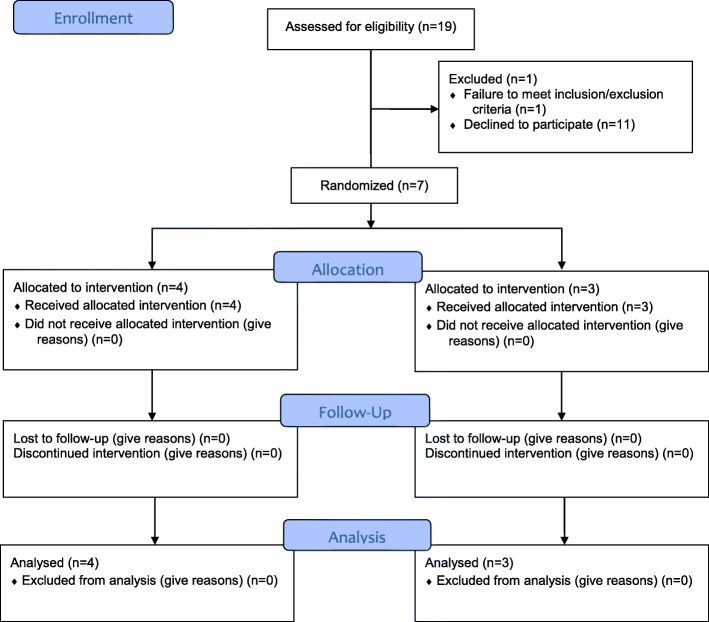
CONSORT flow

From July 2014 to June 2018, a total of 7 patients underwent randomization (Fig. 1). Four patients were randomized to the treatment arm and received UFH, and 3 patients were randomized to receive SOC only. The average age of subjects in the UFH group was 26 ± 3.8 years, range 26 to 33 years. The mean age of subjects in the SOC group was 32 ± 18.5 years, range 18 to 53 years. Three subjects had SCD genotype HbSS, 2 in the UFH arm and 1 in the SOC arm. SCD genotype HbSC was present in 3 subjects, 2 in the UFH arm and 1 in the SOC arm. One subject in the UFH group had SCD genotype HbSβ^0^. Baseline patient characteristics are detailed in Table 4.

**Table 4:** Patient characteristics

	Standard of care (***N*** = 3)	Unfractionated heparin (***N*** = 4)
**Age (years)**	32 ± 18.52	29.25 ± 3.77
	25 (18, 53)	29 (26, 33)
**Sex**		
**Male**	1 (33%)	2 (50%)
**Female**	2 (67%)	2 (50%)
**Race**		
**African American**	3 (100%)	4 (100%)
**Genotype**		
**HbSS**	1 (33%)	2 (50%)
**HbSC**	1 (33%)	2 (50%)
**HbSβ**^**0**^	1 (33%)	0 (0%)
**Prior episode of ACS**	3 (100%)	4 (100%)
**Current hydroxyurea use**	3 (100%)	4 (100%)
**History of VTE**	0 (0%)	0 (0%)
**History of myocardial infarction**	0 (0%)	0 (0%)
**Chronic lung disease**	0 (0%)	0 (0%)
**History of stroke**	0 (0%)	0 (0%)
**Active tobacco use**	2 (67%)	1 (25%)

*n* (%) for categorical variables. Mean ± SD and median (Min, Max) for continuous variables

Abbreviation: *ACS* acute chest syndrome, *VTE* venous thromboembolism

### Standard of care

All subjects received intravenous fluids, antibiotics, and analgesia. Antibiotics included penicillin, macrolides, and vancomycin. Analgesia consisted of acetaminophen, nonsteroidal antiinflammatory drugs, and opioids. All subjects, except for 1 in the SOC group, received supplemental oxygen. Similarly, all subjects, except for 1 in the UFH group, received a red blood cell transfusion. Two subjects, 1 in each arm, received an exchange transfusion.

### Feasibility criteria

The following prespecified feasibility criteria were not met: the capacity to consent eligible individuals and the timely notification of hospitalized patients with ACS. There were no instances of improper administration of the study drug or inability to completely and accurately collect clinical data of interest.

The primary reason for the inability to consent eligible individuals was a patient refusal. Eleven potential subjects refused participation. Reasons for refusal included a lack of interest in study participation (*n* = 9) and concerns about the risk of bleeding with heparin (*n* = 2). Patients’ unfamiliarity with the PI and the clinical trial likely played a role in their decision. Failure to notify the PI of hospitalized patients with ACS in a timely fashion prevented randomization within 24 h of diagnosis. This was in large part due to omission by the treating physician. The exact number of potential subjects missed is difficult to quantify with absolute certainty; however, the discovery of several diagnoses of ACS during and after hospitalization is evidence that this was an issue. Thus, as a result of poor enrollment, the study was terminated early.

### Clinical outcomes

#### Primary clinical outcome

The duration of hospitalization was 152 (− 290, 594) h greater in the UFH arm compared with the SOC arm. The UFH group included 1 subject hospitalized for 595 h (Table 5).

**Table 5:** Effects of unfractionated heparin on acute chest syndrome

Outcome	Standard of care	Unfractionated heparin	Mean difference with 95% CI
**Duration of hospitalization (h)**	127 ± 138	279 ± 268	152 (− 290, 594)
	73 (25, 284)	238 (47, 595)	
**Duration of hypoxemia (h)**	51 ± 45	118 ± 61	66 (− 42, 174)
	73 (0, 81)	127 (47, 168)	
**Duration of fever (h)**	0 ± 0	25 ± 30	25 (− 33, 162)
	0 (0, 0)	20 (0, 59)	
**Duration of leukocytosis (h)**	53 ± 25	118 ± 61	64 (− 33, 162)
	61 (25, 73)	127 (47, 168)	
**Duration of moderate to severe pain (h)**	89 ± 73	118 ± 61	29 (− 100, 158)
	73 (25, 168)	127 (47, 168)	
**Total dose of opioids (mg oral morphine equivalents)**	1167 ± 1113	3447 ± 4466	2280 (− 4651, 9211)
		1786 (317, 9900)	
**Number of red blood cell transfusions**	4	5	
**Transfer to the intensive care unit**	1 (33%)	0 (0%)	
**Mechanical ventilation**	0 (0%)	0 (0%)	
**Multiorgan dysfunction syndrome**	0 (0%)	0 (0%)	

*n* (%) for categorical variables. Mean ± SD and median (Min, Max) for continuous variables

#### Secondary clinical outcomes

The duration of hypoxemia, leukocytosis, fever, and moderate to severe pain was 66 (− 42, 174), 25 (− 20, 70), 64 (− 33, 162), and 29 (− 100, 158) h longer, respectively, in the UFH group compared with the SOC group. Total oral morphine equivalents administered were 3447 ± 4466 and 1167 ± 1113 mg in the SOC and UFH arms, respectively. Subjects in the UFH group received 5 red blood cell transfusions compared with 3 red blood cell transfusions in the SOC group. One subject in the SOC arm required to transfer to the intensive care unit. No subjects required mechanical ventilation or developed multiorgan failure syndrome (Table 5).

#### Safety

One subject in the UFH group had two minor episodes of self-limited epistaxis. No other bleeding, thrombocytopenia, or other adverse events were noted.

## Discussion

We describe the results of a single-center, randomized controlled, open-label, pilot study to determine the feasibility of performing a larger multicenter phase III trial to assess the effects of UFH in ACS. Two of the prespecified feasibility criteria were not met: the capacity to consent eligible individuals and the timely notification of hospitalized patients with ACS necessary to permit randomization within 24 h of diagnosis; thus, as a result of poor enrollment, the study was terminated early.

The duration of hospitalization was 152 (− 290, 594) h greater in the UFH arm compared with the SOC arm. Likewise, the duration of hypoxemia, leukocytosis, fever, and moderate to severe pain was 66 (− 42, 174), 25 (− 20, 70), 64 (− 33, 162), and 29 (− 100, 158) h longer, respectively, in the UFH group compared with the SOC group. While the large effect sizes might indicate UFH may not be beneficial in ACS, the wide confidence intervals are indicative of imprecision due to the small sample size limiting any reliable inferences. No safety concerns were noted during the study with only two episode of self-limited epistaxis noted in 1 subject receiving UFH.

To our knowledge, this was the first clinical trial to evaluate the effects of UFH in ACS; however, a couple of recent studies have evaluated the use of low molecular weight heparin in VOC. One randomized, double-blind, placebo-controlled study of patients with VOC treated with tinzaparin resulted in a significant reduction in the duration of hospitalization, duration of VOC, and the number of days with the most severe pain scores [10]. Another randomized, double-blind, placebo-controlled trial evaluated hypercoagulable markers and pain scores in hospitalized patients with VOC treated with dalteparin and found no difference in hypercoagulable markers but decreased pain scores at days 1 and 3 in subjects receiving dalteparin [11].

Since the study was terminated early due to poor enrollment, it is important to discuss some of the factors responsible, so others are aware of these issues and avoid similar missteps in the future. The PI’s primary clinical responsibility is to care for patients with disorders of hemostasis; thus, the PI is not involved in the day-to-day care of SCD patients. This unfamiliarity with the PI may have made patients more hesitant regarding study participation. In addition, patients were often unaware of the clinical trial prior to being approached in the hospital, while acutely ill and in distress. At that time, patients were expectedly more preoccupied with the acute management of their symptoms rather than discussing the study. Informing patients about the study during their regular outpatient clinic visits may have avoided this situation. Also, issues arose with PI notification of potential subjects. Again, in part, this was related to the PI not being involved in the day-to-day clinical care of SCD patients.

During the course of the study, efforts were made to mitigate some of the above problems. A dedicated research coordinator intimately involved with the care of SCD patients was assigned to the study to decrease the level of unfamiliarity among patients and ensure PI notification of potential subjects, in part, through the use of the EMR. Despite these efforts, enrollment did not improve, so the decision was made to terminate the study. While the study was designed in conjunction with our institution’s sickle cell disease healthcare providers valuable input based on their day-to-day interactions with our sickle cell disease patient population; in retrospect, it would have been beneficial to involve patient representatives and have patients complete a pre-study feasibility survey whether or not they would be willing to participate in the study, if at some point they were deemed a candidate, to better determine if achieving the enrollment goal was possible.

Others have experienced similar difficulties performing clinical trials in sickle cell disease. The IMPROVE trial, a multicenter clinical trial comparing two alternate dosing patient-controlled analgesia strategies for management of VOC-related pain, was terminated early due to slow accrual. Specific problems included communication (failure to notify study personnel about potential subjects) and staffing (inadequate study personnel to enroll subjects at night and on weekends) [12].

## Conclusions

Our single-center, randomized controlled, open-label, pilot study to determine the feasibility of performing a larger multicenter phase III trial to assess the effects of UFH in ACS did not achieve prespecified feasibility criteria, resulting in poor enrollment and early termination, and serves to highlight some of the pitfalls experienced in clinical research in SCD. It did show the use of UFH without any major adverse events in 7 subjects. No future large-scale study is planned.

## Data Availability

All data generated or analyzed during this study are included in this article. The study protocol is available from the corresponding author upon request.

## Abbreviations

ACS: Acute chest syndrome
PI: Principal investigator
SCD: Sickle cell disease
SOC: Standard of care
UFH: Unfractionated heparin
VOC: Vasooclusive crisis
